# Morin hydrate alleviated cisplatin-induced testicular toxicity in rats via modulating NLRP3/NF-κB pathway

**DOI:** 10.1007/s00210-025-04867-5

**Published:** 2025-12-27

**Authors:** Sara Nabil Hosney, Asmaa I. Matouk, Fares E. M. Ali, Gehan Hussein Heeba

**Affiliations:** 1 Pharmaceutical Inspection, General Administration of Pharmacists, Assiut, Egypt; 2 Department of Pharmacology and Toxicology, Faculty of Pharmacy, Minia University, Minia, Egypt; 3 Department of Pharmacology and Toxicology, Faculty of Pharmacy, Al-Azhar University, Assiut, Egypt; 4 Michael Sayegh, Faculty of Pharmacy, Aqaba University of Technology, Aqaba, 77110 Jordan; 5 Department of Pharmacology and Toxicology, Faculty of Pharmacy, Minia National University (MNU), Minia, Egypt

**Keywords:** Cisplatin, Morin hydrate, NF-κB, NLRP3, Testicular toxicity

## Abstract

**Abstract:**

Cisplatin (Cis) has been widely used for treating many types of solid tumors. Despite its clinical effectiveness, Cis has a considerable risk of gonadal damage that may cause infertility. Morin hydrate (MH), a natural bioflavonoid, has been known for its antioxidant and anti-inflammatory effects. Our study aimed to investigate whether pretreatment with MH could protect against Cis-induced testicular toxicity. Thirty-five adult male rats were split into five groups (*n* = 7, each); control group received oral 0.5% CMC for 10 days, MH group received oral MH (100 mg/kg) for 10 days, Cis group was given a single dose of Cis (7 mg/kg, i.p) on day 5, (MH 50 + Cis) and (MH 100 + Cis) groups were pretreated with MH (50 mg/kg) and (100 mg/kg), respectively for 5 days before Cis administration, and then, treatment was continued, with either doses, for further 5 days. At the end of the study, blood and testicular tissues were collected for biochemical and histopathological studies. MH administration mitigated the testicular histopathological changes induced by Cis, increased sperm count and motility, and abrogated the abnormalities in sperm morphology. Further, MH enhanced antioxidant status and suppressed the inflammation via downregulating NF-κB and NLRP3 and inflammatory cytokines expression. Our in vitro study revealed that MH enhanced Cis-induced cytotoxicity against cancer cells, including PC3, MCF7, and HepG2. These findings suggested that MH could be applied in Cis chemotherapy regimens as a possible adjuvant therapy to enhance its effect and prevent Cis-induced testicular damage.

**Graphical Abstract:**

**Figure:**
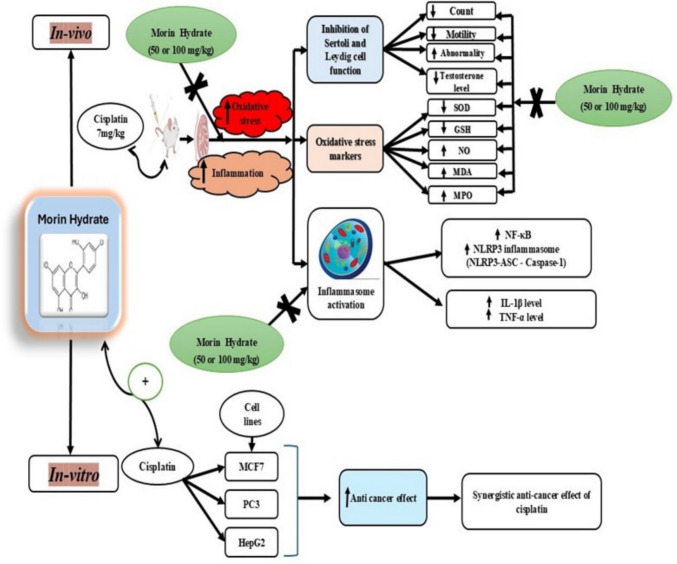
Graphical abstract illustrates the possible protective effects of MH on Cis-induced testicular toxicity

**Supplementary information:**

The online version contains supplementary material available at 10.1007/s00210-025-04867-5.

## Introduction

Cisplatin (Cis), cis-diamine dichloro-platinum II, an antineoplastic agent, has been used effectively for treating endometrial, ovarian, testicular, bladder, head, neck, lung, cervix, and other types of solid tumors (Dasari And Bernard Tchounwou 2014; Sherif et al. 2014; Bostancıeri et al. 2022). The effectiveness of Cis in killing cancer cells is well established. However, its toxicity against normal healthy tissue may necessitate dose reductions or even compromise its clinical efficacy. Among the serious side effects accompanying Cis use are neurotoxicity, nephrotoxicity, and infertility (Zhou et al. 2022; Kandeil et al. 2020). Cis-induced gonadal toxicity arises from its cytotoxic effects on Leydig cells, which are crucial for steroidogenesis, as well as Sertoli cells, which facilitate spermatogenesis (Harman And Richburg 2014). Most patients receiving Cis, in a cumulative dose that exceeds 600 mg/m^2^, experience significantly low androgenic levels, testicular atrophy, oligospermia, or azoospermia (Pont And Albrecht 1997; Zavattaro et al. 2021; Ghezzi et al. 2016). This hypogonadism and failure of spermatogenesis continue for a long period and may be irreversible in some cases. The patient’s quality of life, body image, sexuality, and even the probability of conception are negatively influenced by Cis treatment.

There is a growing interest in oncofertility, which focuses on using adjuvant treatments to preserve fertility in cancer patients given chemotherapeutic agents such as Cis (Chovanec et al. 2017). The precise pathophysiological mechanisms of how Cis can induce testicular toxicity remain unclear. Cis causes DNA adducts formation through its intra- and inter-strand crosslinks with the DNAs’ purine bases, resulting in cell cycle arrest and apoptotic cell death (Rezvanfar et al. 2013; Afsar et al. 2017; Feng-Yang Wang et al. 2018). Moreover, upon binding of Cis to mitochondrial DNA, it disrupts the mitochondrial function and activates NADPH oxidase, leading to a substantial release of reactive oxygen species (ROS), which induce testicular damage (Marullo et al. 2013).

Interestingly, the spermatogenesis process typically occurs under hypoxic conditions, which include ROS production. However, intracellular enzymatic and non-enzymatic antioxidants can neutralize the released ROS. On the other side, Cis-induced oxidative stress exhausts the antioxidant activity and causes disruption of germ and Sertoli cells, leading ultimately to infertility. The testicular damage induced by ROS may occur either directly due to oxidation of lipids, proteins, and DNA or indirectly via ROS-mediated initiation of inflammatory signals (Martin-Hidalgo et al. 2019; Baskaran et al. 2021).

Several studies have documented the beneficial role of natural antioxidants and anti-inflammatory agents in the alleviation of Cis-induced testicular dysfunction (Eid 2016; Shati 2019). Morin hydrate (MH, 3,5,7,2′,4′-pentahydroxyflavone), a member of the flavonoid family, exists in many parts of *Moraceae* plants (Caselli et al. 2016). MH has shown prominent antioxidant and anti-inflammatory features in various disease models that affect the liver, heart, colon, kidney, and testis (Vikash Kumar et al. 2024b; Verma et al. 2024; Touny et al. 2024; Darendelioğlu et al. 2025; Olayinka And Adewole 2021). Additionally, MH could improve sperm qualities and restore testicular activity and testosterone levels in heat-induced testicular damage (Rahul Kumar et al. 2024a) and in diclofenic acid-induced testicular toxicity (Simsek et al. 2025). The anti-inflammatory effects of MH were attributed to the inactivation of nuclear factor kappa B (NF-κB) and NOD-like receptor family pyrin domain containing 3 (NLRP3) inflammasome signaling pathways (Yu et al. 2020). Further, MH could increase cellular antioxidant activities and maintain the oxidant/antioxidant balance in lipopolysaccharide-induced mastitis (Yu et al. 2020). Besides the beneficial antioxidant and anti-inflammatory effects of MH, it possesses cytotoxic activity against cancer cells (Venu Gopal 2013). The present study aimed to investigate whether pretreatment with MH could prevent testicular damage induced by Cis. Further, we explored, in vitro, the impact of MH on Cis-induced cytotoxicity on human breast (MCF7), prostate (PC3), and liver (HepG2) cancer cell lines.

## Materials and methods

### In vivo studies

#### Drugs and chemicals

The drugs and chemicals are as follows: MH powder (Sigma-Aldrich Chemical; St. Louis, MO, USA; Catalog No. M4008), Cis (Mylan S.A.S; Saint-Priest, France), carboxymethyl cellulose (CMC) (El Gomhoria Company; Cairo, Egypt). Rat interleukin 1 beta (IL-1β) ELISA kit (Elabscience Biotechnology; Wuhan, China; Catalog No. E-EL-R0012), rat tumor necrosis factor-alpha (TNF-α) ELISA kit (Elabscience Biotechnology; Wuhan, China; Catalog No. E-EL-R0019), testosterone ELISA kit (Calbiotech Inc.; El Cajon, CA, USA; Catalog No. TE187S-100, RRID: AB_2943692), polyclonal rabbit antibody for NF-κB p65 (Abbexa; Cambridge, UK; Catalog No. abx012874), polyclonal rabbit antibodies for NLRP3, ASC, and caspase-1 (Biospes Co.; Ltd China; Catalog No.YPA1480, Catalog No. YPA1695, and Catalog No. YPA2348), and Monoclonal mouse antibody for β-actin (Elabscience Biotechnology, USA; Catalog No. E-AB-20031, RRID: AB_3662852), respectively.

#### Experimental design

Thirty-five adult male Wistar Albino rats (180–220 g) were obtained from the animal care unit of the Faculty of Veterinary Medicine, Assiut University, Egypt. Before using the animals, they were housed in standard cages for 2 weeks. They were kept in controlled environmental conditions, including a relative humidity range of 45 ± 5%, 25 ± 2 °C, and a 12-h light/dark cycle. The rats were supplied with standard laboratory food and water. The Animal Ethics Committee at the Faculty of Pharmacy, Minia University, accepted all animal care conditions and experimental procedures (Permit Number: 2301102). The experimental design and all other experimental procedures followed the Animal Research Reporting of In Vivo Experiments, 2020 (ARRIVE 2.0) guidelines.

After 2 weeks of acclimatization, 35 male rats were randomly split into five groups (seven per group), as illustrated:

- Control group: rats were given oral 0.5% CMC (0.5 ml/100 g) for 10 days.
- Control MH group: rats were given MH orally (100 mg/kg/day) suspended in 0.5% CMC (1 ml for each rat) for 10 days.
- Cis group: rats were injected with a single dose of Cis (7 mg/kg, i.p) on the 5th day of the experiment (Heeba et al. 2016; Elsherbiny et al. 2016).
- MH 50 + Cis group: rats were given oral MH (50 mg/kg/day) for 5 days. On the 5th day, they were injected with a single dose of Cis (7 mg/kg, i.p). Then, treatment with oral MH (50 mg/kg/day) was continued till day 10 of the experiment.
- MH 100 + Cis group: rats were given oral MH (100 mg/kg/day) for 5 days. On day 5th, they were injected with a single dose of Cis (7 mg/kg, i.p). Then, treatment with oral MH (100 mg/kg/day) was continued till day 10 of the experiment.
- The doses of MH were selected based on our preliminary study and on the ability of MH to combat oxidative stress, inflammation, and apoptosis in different organs, including the liver, kidney, and testes (Li et al. 2019; Sang et al. 2017; Simsek et al. 2025).

#### Blood and testicular tissue collection

After 12-h fasting from the last treatment, blood samples were collected through cardiac puncture technique under ketamine (100 mg/kg/i.p) anesthesia. Serum samples were separated by centrifugation of the blood at 1677 × g for 5 min at 4 °C. Then, serum samples were stored at − 80 °C till the assessment of testosterone levels. The testes were separated, washed, dried with filter paper, and weighed for relative testicular weight calculation. The sperm parameters were assessed using the left cauda. The left testis was dissected into two halves; one half was homogenized and centrifuged at 6708 × g for 10 min at 4 °C; then, the separated clear supernatant was kept at − 80 °C for biochemical markers estimation, including GSH, SOD, MPO, NO, MDA, TNF-α, and IL-1β. The second half was homogenized and stored in a lysis buffer at − 80 °C for western blot analysis of NF-κB and NLRP3 inflammasome expression. The right testes were kept in 10% neutral formalin and prepared for histopathological examination.

#### Analysis of sperm quality

The left cauda epididymis was immediately separated from each rat and minced in 5 ml of phosphate buffer saline (PBS), PH 7.4. Then, incubation was done for 15–20 min at 37 °C to allow sperms to exit from the tubules, forming a suspension. The left cauda suspension was used to record and calculate the sperm motility percentage in at least 5 fields at × 400 magnification using an OPTIKA light microscope. Following the instructions mentioned by Kenjale et al. (2008), the total sperm number could also be evaluated. Sperm concentrate (× 10^6^/ml) and percentage of sperm abnormalities and motility (%) were recorded according to methods described by Ateşşahin et al. (2006) and Sönmez et al. (2005), respectively.

#### Estimation of serum testosterone level

Using a commercially available testosterone ELISA kit, the serum testosterone levels were quantitatively estimated (Albert Chen et al. 1991).

#### Estimation of testicular oxidative stress parameters

In testicular homogenates and following the instructions of the method described by Ellman (1959) and Uchiyama and Mihara (1978), levels of reduced glutathione (GSH) and malondialdehyde (MDA) were determined, respectively. Furthermore, the enzymatic activity of testicular superoxide dismutase (SOD) was estimated using the described techniques by Marklund (1985). The enzymatic activity of myeloperoxidase (MPO) was determined with the method documented by Manktelow and Meyer (1986). Following the technique described by Montgomery and Dymock (1962), the estimation of testicular Nitric oxide (NO) levels was done by assessing the total nitrate/nitrite levels.

#### Estimation of pro-inflammatory cytokines

Testicular TNF-α and IL-1β levels were assessed using commercially available ELISA kits (Ning-Ke Guo et al. 2023; Ezz-Eldin et al. 2024).

#### Western blot analysis

Western blotting (WB) technique has been used according to the method described by Ali et al. (2018). The testicular tissues were homogenized in ice-cold Tris lysis buffer (400 mM NaCl, 0.5% Triton X-100, 50 mM Tris, pH 7.4) with a 1% protease inhibitor cocktail (Biospes, China) at 4 °C for 30 min. Then, homogenates were centrifuged for 10 min at 6708 × g to remove any tissue residuals. The collected supernatants were stored at − 80 °C, and the protein concentrations were determined in the lysates following the previously described method by SF Wang et al. (1996). A volume (60 µg) of protein in each sample was taken and loaded in each lane, and then the volume was adjusted to 10 µl with water for injection. Further, a volume of 10 µl of 2 × Laemmli buffer (Ambion, USA) was added to all tubes, and the mixtures were subjected to denaturation by heating at 95 °C for 5 min before being loaded on the gel. Using 10% SDS-PAGE gel electrophoresis, the protein samples were resolved and then transferred, through semi-dry transfer methods, to polyvinylidene fluoride (PVDF) membrane (Millipore, Merck, USA) (Towbin et al. 1979). After blocking at 37 °C for 60 min with 5% non-fat milk in tris-buffered saline and Tween 20 (TBST), the membranes were incubated overnight with anti-NLRP3 (dilution 1:1000), anti-ASC (dilution 1:1000), anti-cleaved caspase-1 (dilution 1:1000), anti-NF-κB (dilution 1:3000), and anti-β-actin antibody (dilution 1:5000) at 4 °C. After three times of washing, the membranes were incubated with alkaline phosphatase-conjugated secondary antibody (Biospes, China, dilution 1:5000) for 60 min. The BCIP/NBT substrate detection kit (Biospes Co., Ltd, China; Catalog No. BWR1067) was used for band visualization. The bands were analyzed in reference to β-actin with the use of ImageJ® software (National Institutes of Health, Bethesda, USA).

#### Histopathological examination of testicular and epididymal tissues

Testicular tissue samples were taken for histopathological evaluations. The right testes were immediately washed with PBS, kept in 10% neutral formalin, dehydrated with a graded ethanol series, and cleared with xylene. After that, paraffin sections at 4–5 µm thick were processed and stained with hematoxylin and eosin (H&E) (Suvarna et al. 2018). A light microscope (Olympus CX 21) was used to examine these histopathological preparations. The microscopic pathological findings of the testicular tissues have been scored from (−) to (+ + +) following the instructions of the previously described method (Derelanko 2008). This scoring has been illustrated in Table 1. The histopathological examination and scoring of all slides were conducted by a blinded histopathologist.

**Table 1:** Scoring intensity of the histopathological alterations induced by Cis in the testes and the epididymis (H&E)

Histopathological alterations/group	Control	MH (100 mg/kg)	Cis (7 mg/kg)	MH (50 mg/kg) + Cis	MH (100 mg/kg) + Cis
Separation of the spermatogenic cell layers from the basement membranes	−	−	+ + +	+ +	−
Vacuolation of the wall of the seminiferous tubules	−	−	+ + +	+ +	−
Disappearance of the cell layers of the seminiferous tubules	−	−	+ + +	−	−
Vacuolation of the epithelium lining the tubules of the epididymis	−	−	+ + +	−	−

The microscopic pathological findings of the testicular and epididymal tissues were scored based on the intensity of histopathological alterations induced by Cis on a scale from (−) to (+ + +), where (−) is indicated for normal with no significant change, (+ +) is for moderate changes, and (+ + +) is for severe changes

*MH* morin hydrate, *Cis* cisplatin, *H&E* hematoxylin and eosin

### In vitro studies

#### Kits, chemicals, and cancer cell lines

Human prostate (PC3), breast (MCF7), and liver (HepG2) cancer cell lines were obtained from American Type Culture Collection (ATCC) (VA, USA; Catalog No. CRL-1435, Catalog No. HTB-22, and Catalog No. HB-8065, RRID: CVCL_0027), respectively. They were supplemented with 10% fetal bovine serum (FBS) obtained from Hyclone (USA, Catalog No. SH30070.03) and cultured using Dulbecco’s modified Eagle’s media (DMEM) obtained from Invitrogen Life Technologies (Carlsbad, CA, USA, Catalog No. A4192102). (3-[4,5-dimethylthiazol-2-yl]−2,5-diphenyl tetrazolium bromide) or MTT was purchased from Sigma-Aldrich Chemical (St Louis, MO, USA, Catalog No. 298–93-1). In Vitro Toxicology Assay Kit MTT-Based obtained from Sigma-Aldrich Chemical (St Louis, MO, USA, Stock No. TOX1-1KT). The propidium iodide flow cytometry kit for the analysis of the cell cycle was obtained from Abcam (Cambridge, UK, Catalog No. ab139418). Annexin V-FITC Apoptosis Detection Kit was obtained from BioVision (Mountain View, CA, Catalog No. K101-25).

#### Evaluation of the cytotoxicity of Cis, MH, and their combination

The cytotoxicity of the tested drugs was assessed by counting viable cells using the MTT assay. Here, we used this assay to determine the half-maximum inhibitory concentration (IC_50_) for Cis, MH, and their combination (Mosmann 1983). PC3, MCF7, and HepG2 cells were cultured using DMEM and followed the instructions of the previously mentioned assay method by Vistica et al. (1991). In 96-well plates, the cells were seeded and treated with different concentrations (0.1, 1, 10, 50, and 250 µM) of Cis, MH, or (Cis + MH) in triplicate. The purple solutions yielded from the dissolved formazan crystals were spectrophotometrically measured at 570 nm. The IC_50_ (µM) value of the tested drug was calculated, which indicates the concentration at which 50% of the cells had been inhibited (the viability reduced to 50% of the control).

#### Evaluation of Cis, MH, and their combination effect on cell cycle analysis

Through a quantitative DNA content analysis in tissue culture cell lines using a flow cytometry assay, the cell cycle status in these tissues can be detected (Darzynkiewicz et al. 2017). Generate a single-cell suspension of MCF7 cells, wash the formed cell pellet with 1X PBS, treat with Cis, MH, and their combination then fix those cells in 66% ethanol and store for 2 h at 4 °C, transfer the previously prepared cells from 4 °C to room temperature, gently resuspend the cell pellet in 200 µL 1X propidium iodide (PI) + RNase staining solution, and incubate the cells with PI at 4 °C for 20–30 min in the dark to be assessed via flow cytometry analysis.

#### Evaluation of Cis, MH, and their combination effect on apoptosis

The Annexin V-FITC apoptosis detection kit was used to assess the impact of Cis, MH, and their combination on MCF7 apoptosis. The assay depends on the binding of Annexin V, a protein with a high affinity for phosphatidylserine (PS) (Koopman et al. 1994). PS is normally present on the inner face of the cell membrane, but it translocates from the inner face to the outer face of the plasma membrane in the early stage of apoptosis. Once PS is located on the cell surface, it can be easily bound by Annexin V after induction of apoptosis in MCF7 cells by the tested drugs. The cells (1–5 × 10^5^) were collected using centrifugation, resuspended in 1X binding buffer, stained with Annexin V-FITC and PI (optional), and incubated at room temperature for 5 min in the dark. Flow cytometry or fluorescence microscopy was used for the detection of apoptosis.

### Statistical analysis

In GraphPad Prism (Version 8.0 for Windows, GraphPad Software, San Diego, CA, USA), the results were analyzed and statistically calculated. Using one-way analysis of variance (ANOVA) followed by the Tukey–Kramer post-analysis test for multiple comparisons for analysis of significant differences between groups. The results were considered statistically significant at a *p*-value (< 0.05), and the values were expressed as mean ± standard deviation (SD).

## Results

### In vivo results

#### Effect of MH on Cis-induced changes in relative testicular weight, sperm parameters, and serum testosterone level

**Fig. 1:**
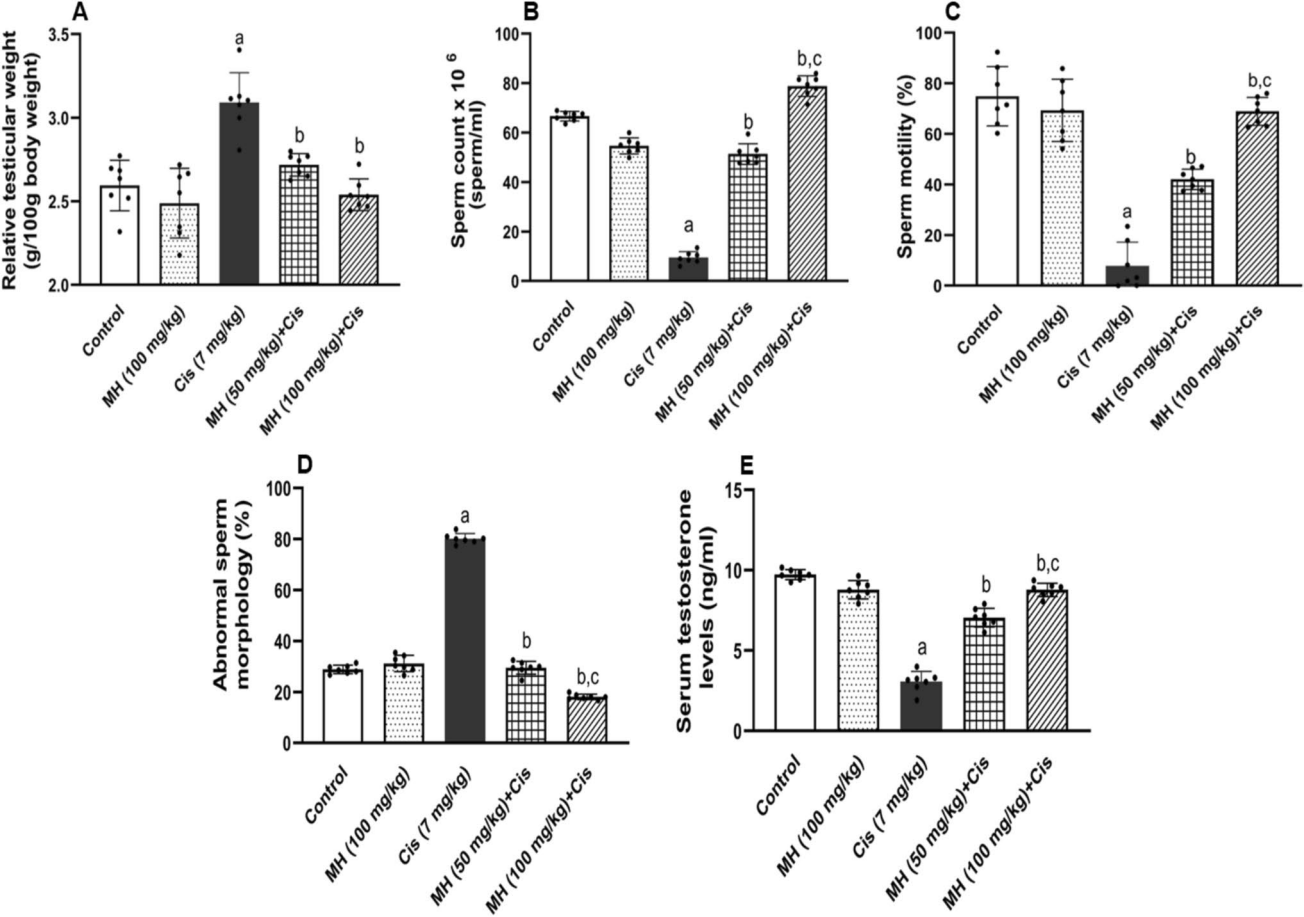
Effect of treatment with oral MH (50 mg/kg or 100 mg/kg) on Cis-induced changes in **A** relative testicular weight, **B** sperm count × 10^6^, **C** sperm motility%, **D** abnormal sperm morphology%, and **E** serum testosterone level. Each value was expressed as mean ± SD of seven rats per group. (a), (b), and (c) indicate significant differences at (*p* < 0.05) from control, Cis, and MH 50 + Cis groups, respectively

Compared to the control group, a significant increase (*p* < 0.05) in the relative testicular weights of the Cis group was observed. However, pretreatment with MH either 50 mg/kg or 100 mg/kg resulted in a significant (*p* < 0.05) decrease in the relative testicular weights (Fig. 1A). Notably, there were no remarkable changes between the control MH (100 mg/kg) and the control group. In addition, in Cis-treated rats, when compared to control rats, we observed a marked decrease (*p* < 0.05) in sperm count and sperm motility as well as a significant increase (*p* < 0.05) in the percentage of sperm with abnormal sperm morphology (Fig. 1B, C, and D), respectively. However, pretreatment with MH in either doses (50 mg/kg or 100 mg/kg) significantly increased the sperm count (*p* < 0.05) (Fig. 1B), enhanced the sperm motility (*p* < 0.05) (Fig. 1C), and markedly reduced the percentage of morphologically abnormal sperms (*p* < 0.05) (Fig. 1D). Compared with MH (50 mg/kg), the sperm qualities were markedly (*p* < 0.05) improved upon using MH (100 mg/kg). The serum testosterone levels of the Cis-treated rats were significantly (*p* < 0.05) declined compared to control rats. However, rats treated with MH exhibited a significant (*p* < 0.05) elevation in serum testosterone levels compared to Cis-treated rats (Fig. 1E).

#### Effect of MH on Cis-induced oxidative stress

**Fig. 2:**
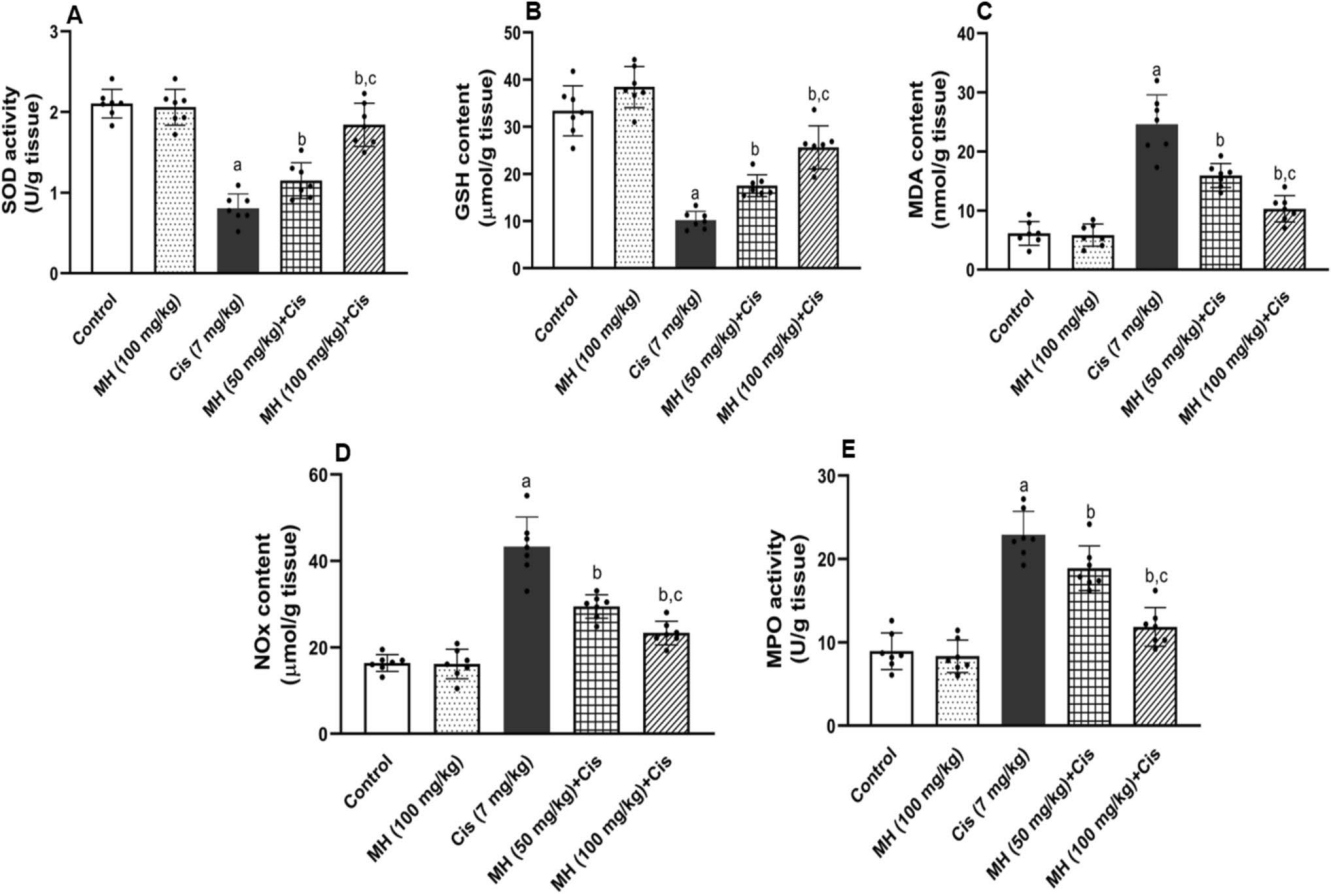
Effect of treatment with oral MH (50 mg/kg or 100 mg/kg) on Cis-induced changes in testicular: **A** superoxide dismutase (SOD), **B** reduced glutathione (GSH), **C** malondialdehyde (MDA), **D** nitric oxide (NO), and **E** myeloperoxidase (MPO). Each value was expressed as mean ± SD of seven rats per group. (a), (b), and (c) indicate significant differences at (*p* < 0.05) from control, Cis, and MH 50 + Cis groups, respectively

In comparison with control rats, the testicular homogenates of Cis-treated rats exhibited a significant (*p* < 0.05) decline in the enzymatic activities of SOD and GSH (Fig. 2A and B), respectively. In contrast, MDA, NO levels, and MPO activity were significantly (*p* < 0.05) elevated (Fig. 2C, D, and E), respectively. These findings indicated an increased oxidative stress in the Cis group when compared to the control group. In contrast, the MH-treated rats in either doses 50 mg/kg or 100 mg/kg showed a significant elevation in the antioxidant biomarkers: SOD (*p* < 0.05) and GSH (*p* < 0.05) (Fig. 2A and B), respectively. Further, treatment with MH significantly abrogated the Cis-induced elevation in testicular levels of MDA (*p* < 0.05), NO (*p* < 0.05), and MPO activity (*p* < 0.05), as shown in (Fig. 2C, D, and E), respectively. Notably, the alleviation of Cis-induced oxidative stress was prominent (*p* < 0.05) in the group that received the higher MH dose. There were no remarkable changes in oxidative stress parameters between the control MH (100 mg/kg) and the control group.

#### Effect of MH on Cis-induced testicular inflammation

**Fig. 3:**
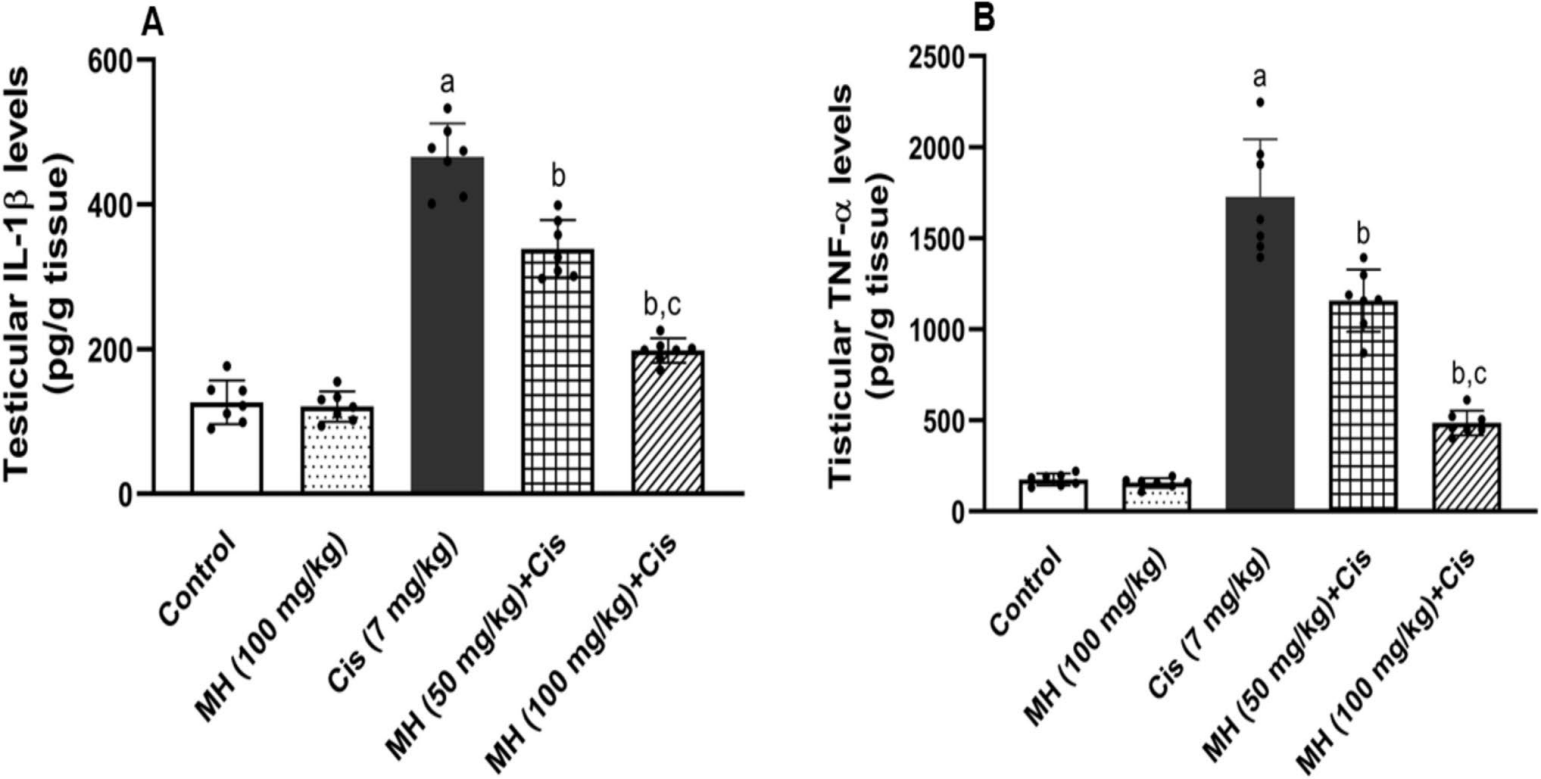
Effect of treatment with oral MH (50 mg/kg or 100 mg/kg) on Cis-induced changes in testicular: **A** interleukin 1β (IL-1β) and **B** tumor necrosis factor-alpha (TNF-α). Each value was expressed as mean ± SD of seven rats per group. (a), (b), and (c) indicate significant differences at (*p* < 0.05) from control, Cis, and MH 50 + Cis groups, respectively

**Fig. 4:**
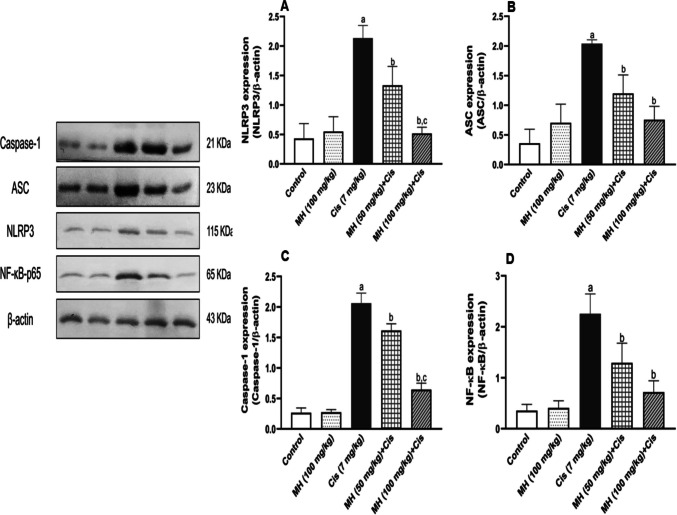
Effect of treatment with oral MH (50 mg/kg or 100 mg/kg) on Cis-induced changes in testicular: **A** NLRP3, **B** ASC, **C** caspase-1, and **D** NF-κB. The expression was determined by Western blot analysis, in which β-actin was used as a control, and the densitometric analysis of the bands was also shown. Each value was expressed as mean ± SD. (a), (b), and (c) were referred to as significantly different at (*p* < 0.05) from control, Cis, and MH 50 + Cis groups, respectively

In the Cis group, the testicular IL-1β and TNF-α content were significantly (*p* < 0.05) upregulated compared to those of control rats (Fig. 3A and B), respectively. Alternatively, MH treatment (50 or 100 mg/kg) attenuated this inflammatory response, as observed by the significant decline in both testicular IL-1β (*p* < 0.05) and TNF-α (*p* < 0.05) levels (Fig. 3A and B), respectively. Furthermore, significant (*p* < 0.05) upregulations of testicular NLRP3, ASC, Caspase-1, and NF-κB protein expressions were reported in Cis-administered rats compared to those of control rats (Fig. 4A, B, C, and D), respectively. However, MH administration (50 mg/kg or 100 mg/kg) significantly downregulated the testicular expression of NLRP3 (*p* < 0.05), ASC (*p* < 0.05), caspase-1 (*p* < 0.05), and NF-κB (*p* < 0.05) as represented in (Fig. 4A, B, C, and D), respectively. The higher MH dose showed more prominent effects compared to MH (50 mg/kg). There were no remarkable changes between the control MH and control groups regarding the levels of the inflammatory mediators. It is important to mention that β-actin expression differed between groups. Although housekeeping proteins are commonly used for normalization, their expression can vary under experimental or pathological conditions. In our dataset, the observed variability may represent a limitation on the conclusions drawn from the immunoblot analyses.

#### Effect of MH on testicular histopathological changes induced by Cis administration

**Fig. 5:**
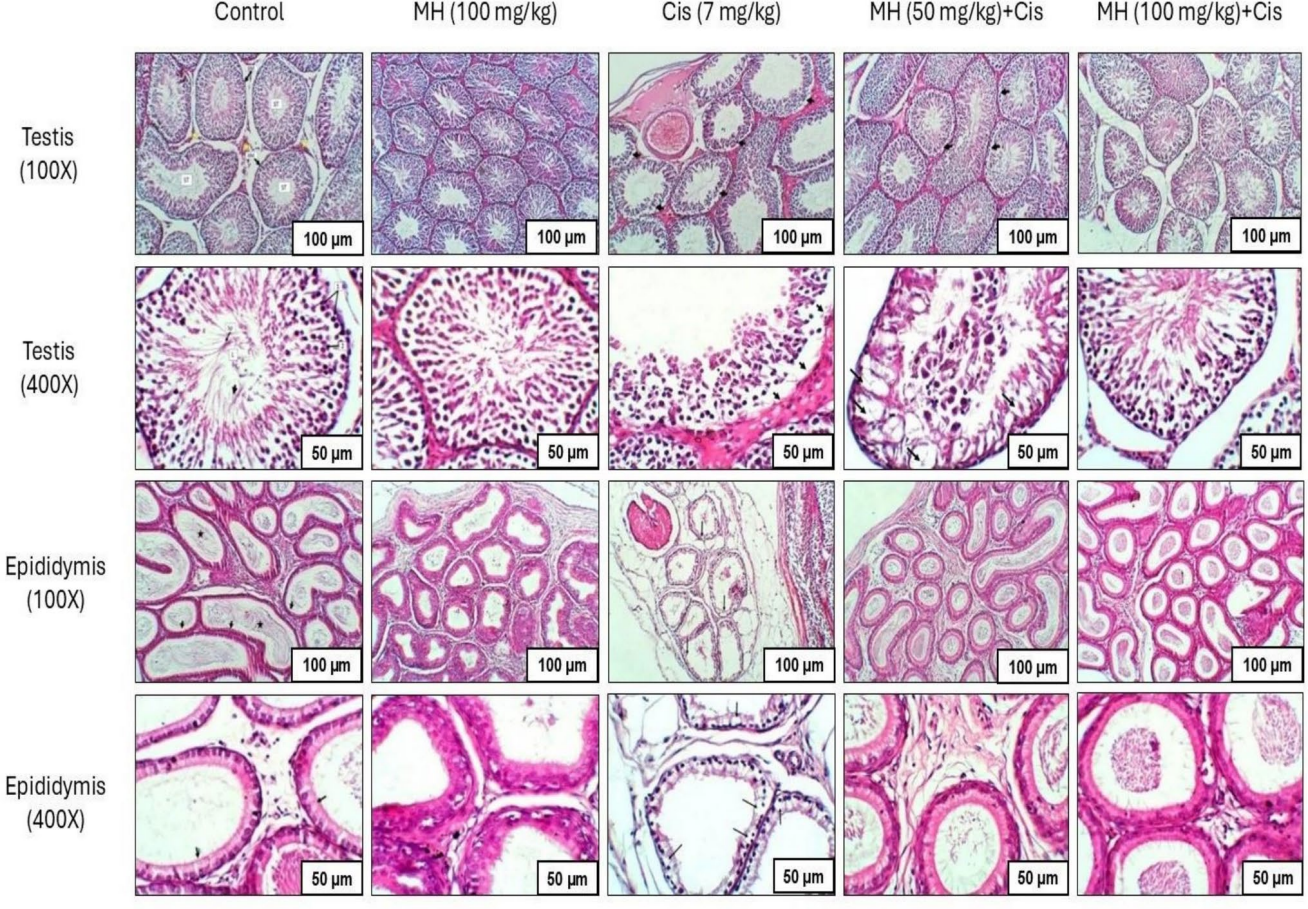
Effect of treatment with oral MH (50 mg/kg or 100 mg/kg) on Cis-induced testicular and epidydimal tissue damage. The figure shows photomicrographs of testicular and epididymal tissues. Testis sections were stained with hematoxylin and eosin (H&E) from all groups and microscopically examined at × 100 and × 400. Testicular sections of the control group and control MH group (100 mg/kg), respectively, show the normal histopathological appearance of the testis with regular contoured seminiferous tubules (ST) lying on the basement membrane (star). Sections of the Cis group showed marked vacuolation of the wall of seminiferous tubules and separation of spermatogenic cell layers from the basement membranes of the seminiferous tubules (arrow). Testicular sections of the MH (50 mg/kg) + Cis group showed a slight separation of spermatogenic cell layers from the basement membranes of the seminiferous tubules (arrow) and well-expressed vacuolation of the wall of seminiferous tubules. Testicular sections of the MH (100 mg/kg) + Cis group showed no evidence for separation or vacuolation of the seminiferous tubules. On the other hand, epididymal sections were stained with (H&E) from all groups and microscopically examined at × 100 and × 400. Epididymal sections of the control group and control MH group (100 mg/kg), respectively, showed the normal histopathological appearance of the epididymis with normal convoluted tubules lined by pseudostratified epithelium (arrow) and filled by spermatozoa (*), whereas sections of Cis control group showing vacuolation of the tubules’ lining epithelium (arrow); however, the epididymal sections of MH (50 mg/kg) + Cis group showed more or less the normal appearance of the tubules and epididymal sections of MH (100 mg/kg) + Cis group showed well expressed healthy tubules with its lining epithelium

In the control group and control MH group, the histopathological examination of testicular and epididymal sections exhibited well-organized contoured seminiferous tubules lying on the basement membrane with regular connective tissue (Fig. 5). Also, the epididymis of the control group and control MH group displayed convoluted tubules that were lined by pseudostratified epithelium (arrow) and were filled by spermatozoa (Fig. 5). In contrast, rats received Cis (7 mg/kg) showed a significant deterioration of testicular tissue that appeared as a well-expressed separation of spermatogenic cell layers from the basement membranes of the seminiferous tubule. Moreover, vacuolation of the seminiferous tubules and a total disappearance of most of the spermatogenic cell layer were observed. Additionally, primary and secondary spermatocytes were also noticed (Fig. 5). Degenerative changes of the epididymis, expressed by vacuolation, were also observed in Cis group (Fig. 5). The light microscopical examination of the seminiferous tubules of the MH (50 mg/kg) + Cis group showed a slight separation of the spermatogenic cell layers from the basement membrane and a well-expressed vacuolation of these tubules was also noticed (Fig. 5). The epididymis had shown a slight change in the normal appearance of the tubules (Fig. 5). Alternatively, the MH (100 mg/kg) + Cis group showed no evidence of separation or vacuolation of the seminiferous tubules (Fig. 5) and tubules of the epididymis also showed normal epithelial lining (Fig. 5).

### Effect size analysis

The effect size indicator is considered a statistical value that quantifies the strength of a relationship between variables. Reporting effect size alongside statistical significance (*p*-values) provides a more complete picture of research findings. Here, we used a one-way ANOVA test to compare between groups; thus, we calculated (*η*2) values as an indicator of the effect size to evaluate the magnitude of differences among groups. All evaluated parameters demonstrated large effect sizes, indicating strong and meaningful differences among the study groups as shown in Table 2.

**Table 2:** Effect size (*η*2) values for the measured parameters

Parameter	*η*^2^ value
Relative testicular weights	0.7096
Sperm count	0.9833
Sperm motility	0.8970
Abnormal sperm morphology	0.9914
Serum testosterone	0.9608
SOD	0.8709
GSH	0.8888
MDA	0.8738
NO	0.8847
MPO	0.8688
IL-1β	0.9504
TNF-α	0.9412
NLRP3	0.9183
ASC	0.8907
Caspase-1	0.9857
NF-κB	0.9063

### In vitro studies

#### Effect of MH-Cis combination on cytotoxicity

**Fig. 6:**
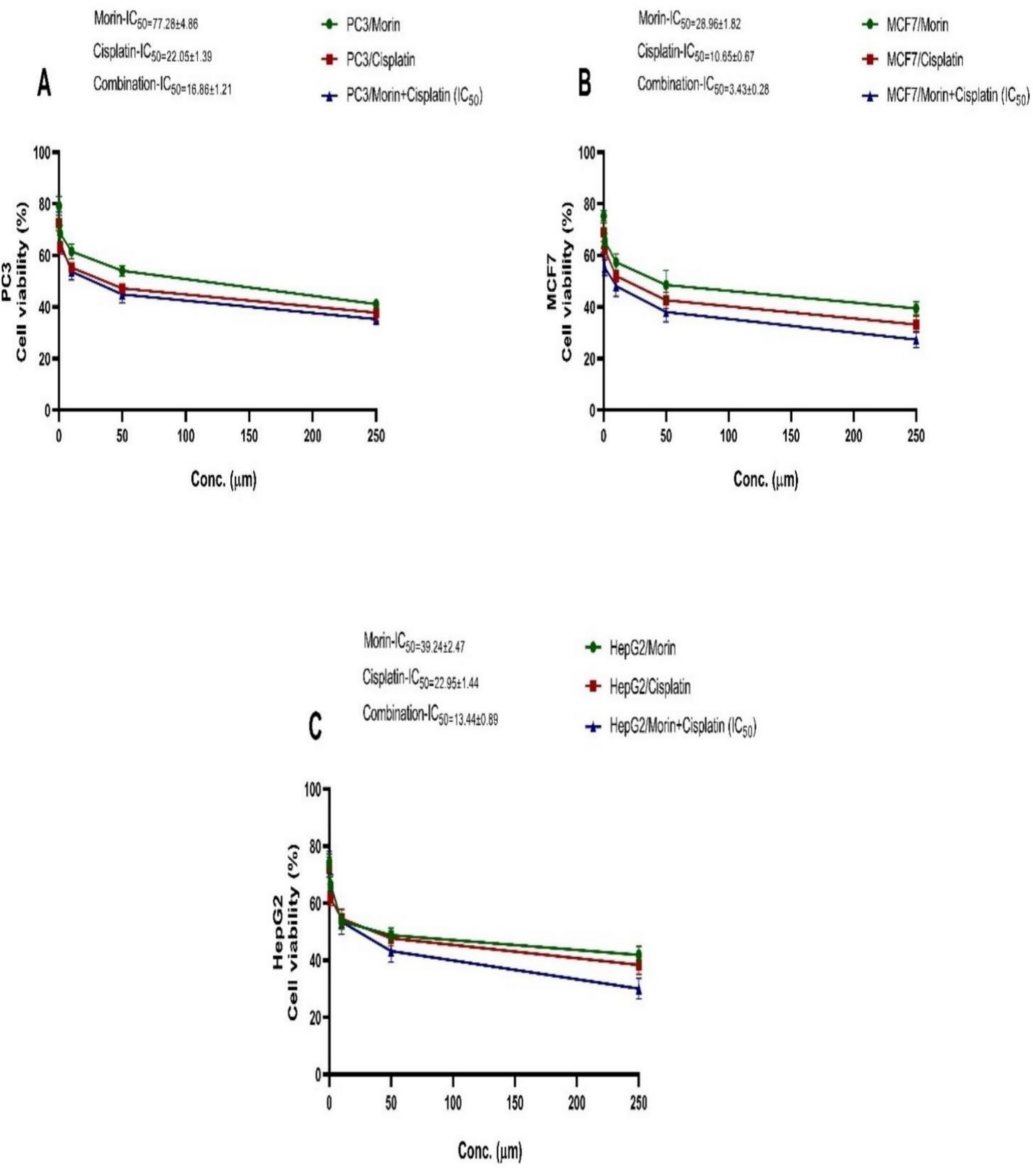
The MTT cell viability assay showing the cytotoxicity of Cis, MH, and their combination in **A** the human prostate (PC3), **B** breast (MCF7), and **C** liver (HepG2) cancer cell lines. In this dose–response curve, the average percentage viability was plotted on the *y*-axis against the logarithm of the concentration for each treatment on the *x*-axis, and the IC_50_ (the concentration corresponding to the 50% viability) was calculated

In human prostate (PC3), breast (MCF7), and liver (HepG2) cancer cell lines, the cytotoxicity of Cis, MH, and their combination was assessed using the MTT cell viability as illustrated in (Fig. 6A, B, and C), respectively. Treatment of these cells with varied concentrations of Cis resulted in a cytotoxic effect with IC_50_ values of 22.05 ± 1.39, 10.65 ± 0.67, and 22.95 ± 1.44 in PC3, MCF7, and HepG2, respectively, whereas MH cytotoxic effect was of IC_50_ values 77.28 ± 4.86, 28.96 ± 1.82, and 39.24 ± 2.47 in PC3, MCF7, and HepG2, respectively. Notably, their combination exhibited a prominent cytotoxic effect with IC_50_ values of 16.86 ± 1.21, 3.43 ± 0.28, and 13.44 ± 0.89 concerning PC3, MCF7, and HepG2 cells, respectively.

It is important to mention that the combination of Cis with MH had a positive impact on the cytotoxic activity of Cis, which was observed by the decline in the values of IC50 of Cis from 22.05 to 16.86 ± 1.21 µM regarding PC3 cells as well as the decline in IC_50_ of Cis regarding MCF7 cells from 10.65 to 3.43 ± 0.28, while in HepG2 cells. The combination of Cis and MH treatment significantly boosted the anticancer effect of Cis, and this was evident by the decrease in the values of IC50 of Cis from 22.95 to 13.44 ± 0.89 µM. From these previously mentioned results, we showed that MH can markedly enhance the cytotoxic activity of Cis.

#### Effect of MH-Cis combination on cell cycle analysis

**Fig. 7:**
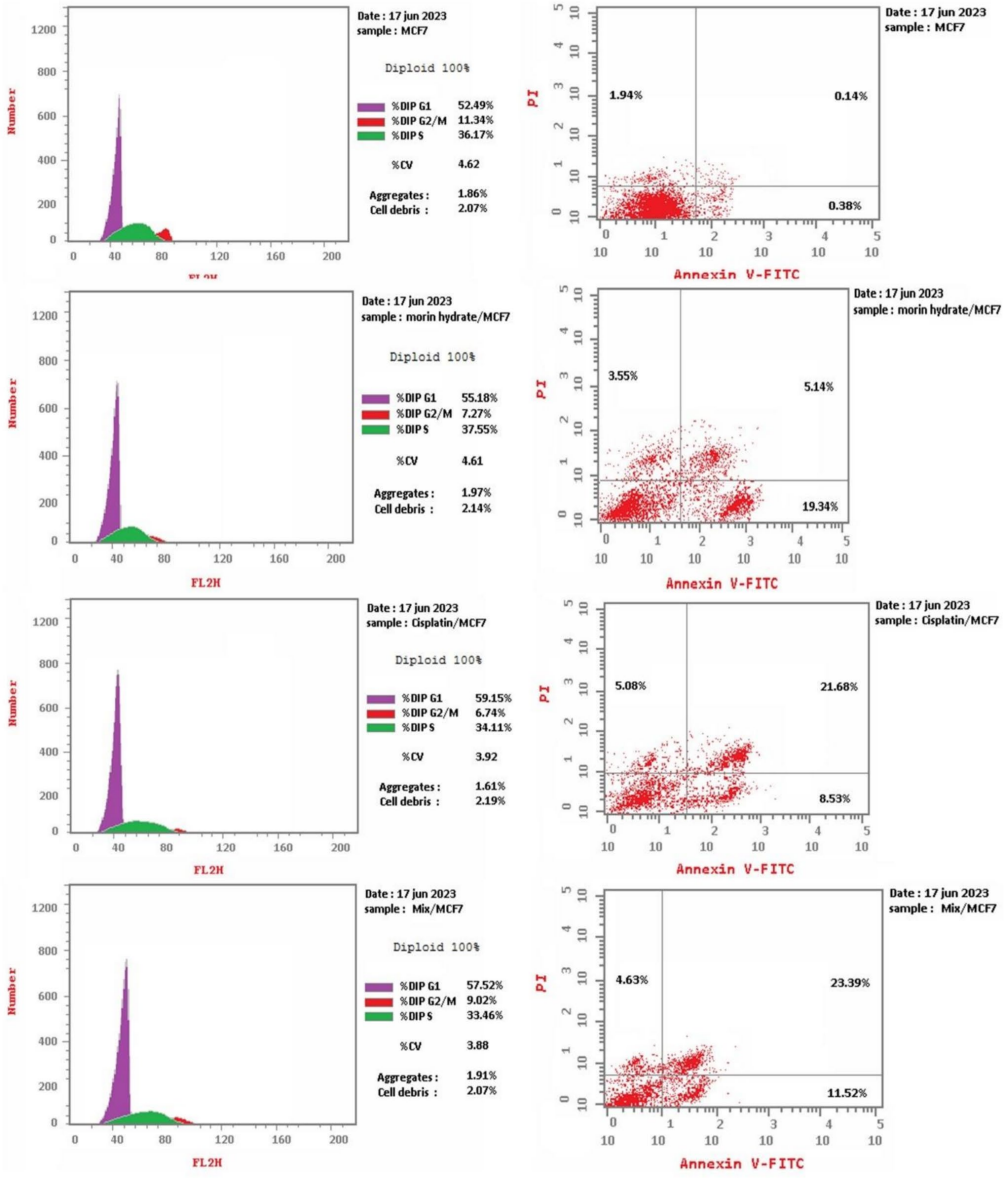
Propidium iodide followed by flow cytometry and Annexin V-FITC assay represented the impact of Cis, MH, and their combination on the cell cycle and apoptosis rate in MCF7 cells. Here, the results were illustrated in the histogram and dot plots

In the present study, the normal cell cycle profile was affected by the treatment of MCF7 cells for 24 h with the IC50 of Cis (10.65 µM) or MH (28.96 µM) and their combination (3.43 µM), respectively, as indicated by the induction of apoptosis. In this regard, the percentage of G0-G1 phase cells markedly increased by 59.15, 55.18, and 57.52-fold, respectively, compared to MCF7 control after 24 h of incubation, as illustrated in the histogram (Fig. 7).

#### Effect of MH-Cis combination on apoptosis

Detection of the apoptotic effect was done through the Annexin V-FITC assay after treatment of MCF7 cells for 24 h with the IC50 of Cis or MH, or their combination. Our data showed early apoptotic and late apoptotic (the lower right and upper right quadrant, respectively, of the dot plots in Fig. 7), which indicated that Cis and MH displayed a significant induction of early and late phases of apoptosis in MCF7 cells. Apoptosis of the control cells was 2.46%. However, treatment with Cis, MH, or their combination led to an average of 35.29%, 28.03%, and 39.54%, respectively, of cells undergoing apoptosis (Fig. 7).

## Discussion

The current study, for the first time, has shown that MH could ameliorate the testicular damage caused by Cis in the adult male rat model. At the same time, our in vitro study revealed that MH potentiated the anticancer effect of Cis on different cancer cell lines, including PC3, MCF7, and HepG2. These findings throw light on the potential use of MH as a promising adjuvant therapy with Cis to reduce its toxicity on the reproductive system without impairing its anticancer properties. Here, the administration of Cis (7 mg/kg) in male rats led to extensive damage to the reproductive system. This was observed by the apparent increase in the testicular weight and the testicular relative weight index. Further confirmation was done by the histopathological examination of testicular and epididymal tissues, which revealed disruptions in the spermatogenic cell layers, vacuolation of spermatocytes and epididymal cells, and disorganization in the seminiferous tubules.

These cytotoxic effects of Cis were accompanied by a marked testicular dysfunction that was evident by the decline in serum testosterone levels. Previous animal and human studies linked the Cis-induced hypogonadism to the damage of Leydig cells and the suppression of steroidogenic acute regulatory protein (STAR), a key regulator of steroidogenesis (Manna et al. 2016). Moreover, Cis-induced destruction of germinal epithelial and Sertoli cells initiates changes in the sperm qualities. Our results, in agreement with others, have documented that Cis induced a marked disruption in the sperm number, motility, and morphology (Abdel-Latif et al. 2022; Keshta et al. 2023; Hamza et al. 2016).

Pretreatment with MH, the natural antioxidant flavanol, successfully abrogated the Cis-induced testicular dysfunction and restored testosterone levels. Further, MH increased sperm count, enhanced its motility, and reduced the percentage of morphologically disturbed sperm. Thus, MH caused an overall improvement in the sperm qualities. Notably, the protective reproductive features of MH have been documented in earlier studies, including those against acrylamide-induced testicular toxicity (Kucukler et al. 2020), procarbazine-induced reproductive damage in rats (Olayinka et al. 2019), and most recently against diclofenac-induced testicular toxicity (Simsek et al. 2025).

Even though DNA damage is the primary blamed factor for Cis-induced testicular cytotoxicity, increased oxidative stress, due to the imbalance between ROS production and the antioxidant status, majorly contributes to the destruction of sperms and the elevated infertility rates (Bansal And Bilaspuri 2011). Based on previous evidence, Cis increases oxidative stress via several pathways, including damaged DNA, upregulation of NADPH oxidase, activation of nitric oxide synthase, and compromised mitochondrial antioxidant defense mechanisms (Choi et al. 2015; Soni et al. 2016; Tse-En Wang et al. 2020). In agreement with these studies, Cis administration increased testicular MPO, one of the peroxidase family released by neutrophils that produce reactive free radicals (Köroğlu et al. 2019). Further, MDA, the product of lipid oxidation by free radicals, was highly formed in the testicular tissues of the Cis group. Since testes, particularly spermatozoa, are rich in polyunsaturated fatty acids, exposure to ROS destroys these lipids and forms MDA adducts. These adducts have cytotoxic effects by binding to DNA and proteins, leading to severe sperm damage (Lenzi et al. 2000; Saral et al. 2016).

Another contributor to the Cis-induced testicular damage was the elevated NO levels which react with superoxide radicals forming the highly oxidizing agent, peroxynitrite. We reported a rise in NO levels in testicular tissues that were correlated to the overexpression of testicular iNOS. While iNOS levels are typically low, they become highly expressed upon exposure to stressful conditions such as excess ROS or inflammatory mediators (Matouk et al. 2022; Famurewa et al. 2020). As a result of oxidative and nitrosative stress induced by Cis, our findings reported a marked depletion in the levels of the antioxidant GSH and SOD (Othman et al. 2023; Anand et al. 2015; Almeer And Abdel Moneim 2018). Conversely, combining MH with Cis ameliorated the elevated testicular NO, MDA, and MPO levels while enhancing the antioxidant status by restoring GSH and SOD levels. The polyphenolic structure of MH is responsible for its potential ROS-scavenging activity (Wolfe And Liu 2008). Also, evidences have reported that MH could inhibit excess NO production and augment the antioxidant enzyme activities through activation of the Nrf2/ARE pathway (Hsieh et al. 2023; Altyar et al. 2023; Rajput et al. 2021).

Continuing previous studies proved that oxidative stress and inflammation are both implicated in Cis-induced testicular damage. The rise in oxidative stress activates NF-κB and activator protein-1(AP-1), leading to an enhanced proinflammatory gene expression (Arab et al. 2024). On the other hand, the activated inflammatory cells, at the inflammation site, produce numerous reactive species, causing a marked exacerbation of oxidative stress (Hussein And Kamel 2023; Lingappan 2018; Sarada et al. 2008). Here, Cis augmented the activation of the nuclear factor-kappa B (NF-κB) pathway, the key regulator of the inflammatory response. Upon NF-κB activation, it translocates to the nucleus and enhances the expression of other proinflammatory cytokines, including TNF-α and IL-1β (Matouk et al. 2023; Qing Guo et al. 2024; Giridharan And Srinivasan 2018). Similarly, upregulation of iNOS and its corresponding increase in NO levels is promoted by NF-κB (Jalal And Kone 2006). Altogether, the testicular damage observed in the Cis group is likely attributed to the rise in inflammatory mediators and oxidative stress.

Inflammasome is a new term that was first introduced by Martinon et al. (2002), referring to cytosolic multiprotein complexes. One of these inflammasomes is the NOD-like receptor family pyrin domain containing 3 (NLRP3) which represents an essential part of the innate immunity (Taniguchi And Karin 2018). NLRP3 is composed of a sensor (NLRP3), an effector (pro-caspase-1), and the adaptor molecule apoptosis-associated speck-like protein which contains a caspase recruitment domain (ASC) (Chang et al. 2015). The NLRP3 combines with the ASC adaptor, leading to the conversion of pro-caspase to caspase-1. The latter promotes further IL-1β secretion and induces pyroptosis which is a form of inflammatory cell death (Zhong et al. 2016; Haitao Guo et al. 2015). Activation of NLRP3 led to the exacerbation of numerous inflammatory diseases, such as neurological disorders, endotoxic shock, acute lung injury, and atherosclerosis (Song et al. 2017; Zhang et al. 2017). Likewise, testicular tissues of Cis-treated rats, in response to the overexpressed NF-κB and inflammatory mediators, exhibited an upregulation of NLRP3/ASC/caspase-1 levels leading to testicular cell pyroptosis and massive inflammatory response (Shaaban et al. 2023; Xiujin Li and Zhong 2014; Akaras et al. 2024). Conversely, MH mitigated this testicular inflammation by attenuating the protein expression of NF-κB and decreasing the TNF-α and IL-1β levels when administered in Cis-intoxicated rats owing to its antioxidant and anti-inflammatory features. Furthermore, MH inactivated the NLRP3/ASC/caspase-1 signaling pathway (Tianzhu et al. 2014; Arjsri et al. 2025). Our findings regarding the anti-inflammatory effects of MH were aligned with the previously documented studies including those against Cis-induced renal and hepatic toxicity as well as in other inflammatory models (Wei et al. 2015; Cai et al. 2018; Verma et al. 2019). All these beneficial effects contributed to the MH preventive effect against Cis-induced testicular toxicity. MH alleviated the Cis-induced testicular damage in a dose-dependent manner since the beneficial effects of MH were more prominent in MH (100 mg/kg) than in MH (50 mg/kg). It is noteworthy to mention that the expression of NLRP3, ASC, caspase-1, and NF-κB was normalized to β-actin which showed modest variability between groups. Such variation in the housekeeping band may have affected the calculated normalized values, leading to a potential under- or overestimation of the exact magnitude of the group differences. Therefore, the Western blot data should be interpreted as semi-quantitative, and the precise relative expression changes in protein expression should be viewed with caution. Nevertheless, the overall pattern of changes (cisplatin-induced upregulation and its attenuation by MH) is consistent with the histopathological findings and the biochemical markers of inflammation and oxidative stress, which together support the conclusion that MH could attenuate Cis-induced testicular injury.

Our in vitro results revealed that MH could act as a promising anticancer adjuvant when used alongside Cis. In the MTT assay, the combination of MH with Cis exhibited a synergistic cytotoxic effect on PC3, MCF7, and HepG2 cells, which was more pronounced than Cis alone. Although the cytotoxic activity of MH has been evidenced in various human cancer cell lines, the precise underlying mechanism is not yet understood (Perumal et al. 2017; Rui Chen and Zhang 2019; Ji et al. 2018). Besides the anticancer activity of MH, the apoptotic effect of MH was evaluated in MCF7 cells, which further highlights its anticancer potential since MH augmented the apoptotic effect of Cis in MCF7 cells.

The present study revealed the potential protective effects of MH against Cis-induced testicular toxicity. However, it included some limitations that can be addressed in future studies such as the lack of using pharmacological inhibitors or siRNA knockdown to confirm the role of NLRP3/ASC/caspase-1 signaling pathway in mediating MH protective effects. Detailed studies are needed to further explore the protective mechanisms of MH against Cis-induced damage of Sertoli and Leydig cells which are responsible for sperm production and fertility. Further, the observed variability in β-actin represents a limitation of the study and introduces some uncertainty into the precise quantitative interpretation of the Western blot results.

## Conclusions

MH, a natural antioxidant polyphenolic agent, has promising therapeutic potential that could mitigate the Cis-induced testicular dysfunction in rats. MH attenuated the Cis-induced elevation in testicular oxidative stress as well as inactivated the NLRP3/ASC/caspase-1 inflammatory and pyroptotic signaling pathways, leading to an improvement in serum testosterone levels and an enhancement in the sperm qualities. Furthermore, MH synergistically impacted the cytotoxicity of Cis on cancer cells. This study suggested that MH could attenuate the Cis-induced testicular damage without opposing its anticancer activities.

## Supplementary information

Below is the link to the electronic supplementary material.

ESM 1 (XLSX 11.4 KB)

## Data Availability

All source data for this work (or generated in this study) are available upon reasonable request.
